# Ruptura esplénica espontánea en malaria complicada por *Plasmodium vivax*

**DOI:** 10.7705/biomedica.7826

**Published:** 2026-08-20

**Authors:** José Ziade-Benítez, María Trinidad Orozco, Mario J. Olivera, Édgar Parra, Julio César Padilla

**Affiliations:** 1 Red de Gestión de Conocimiento, Investigación en Innovación en Malaria, Instituto Nacional de Salud, Bogotá, D. C., Colombia Instituto Nacional de Salud Instituto Nacional de Salud Bogotá, D. C. Colombia; 2 Vigilancia Epidemiológica, Secretaría Departamental de Salud, Guaviare, Colombia Secretaría Departamental de Salud Secretaría Departamental de Salud Guaviare Colombia; 3 Grupo de Parasitología, Instituto Nacional de Salud, Bogotá, D. C., Colombia Instituto Nacional de Salud Instituto Nacional de Salud Bogotá, D. C. Colombia; 4 Grupo de Patología, Instituto Nacional de Salud, Bogotá, D. C., Colombia Instituto Nacional de Salud Instituto Nacional de Salud Bogotá, D. C. Colombia

**Keywords:** Plasmodium, Plasmodium vivax, malaria, Plasmodium, Plasmodium vivax, malaria

## Abstract

Se presenta un caso de ruptura esplénica patológica asociada a malaria complicada por *Plasmodium vivax* en un paciente de sexo masculino, de 28 años, procedente de El Retorno (Guaviare). El paciente fue hospitalizado con fiebre, dolor en el hemitórax izquierdo, vómito, diarrea, taquicardia, taquipnea, hipotensión y signos de inestabilidad hemodinámica, asociados con un estado de choque hipovolémico. Al ingreso, se le administró tratamiento analgésico intramuscular con 500 mg de dipirona y 100 mg de tramadol, y líquidos intravenosos.

En el hemograma se encontró neutrofilia y plaquetopenia, y la gota gruesa fue positiva para *P. vivax* con 24 000 parásitos por μl. Durante su estancia hospitalaria -que se prolongó por solo 4 horas-, el paciente experimentó un deterioro progresivo de su estado general, que culminó en paro cardiorrespiratorio que no respondió a las maniobras de reanimación y resultó en su fallecimiento.

En el estudio histopatológico de bazo se evidenciaron agregados de neutrófilos polimorfonucleares y plasmocitosis. El paciente no alcanzó a recibir tratamiento antimalárico parenteral. La ruptura esplénica asociada a la malaria es una complicación grave y potencialmente mortal. Por ello, toda esplenomegalia dolorosa en un episodio agudo de malaria debe considerarse como un signo de alarma de posible ruptura esplénica.

Es esencial fortalecer la sensibilización de los equipos médicos locales sobre esta complicación, promoviendo el diagnóstico y el tratamiento oportunos para mejorar los desenlaces clínicos.

## Abstract (en)

A case of pathological splenic rupture associated with malaria complicated by *Plasmodium vivax* is presented in a 28-year-old male patient from El Retorno (Guaviare). The patient presented with fever, left side chest pain, vomiting, diarrhea, tachycardia, tachypnea, and signs of hemodynamic hypotension associated with hypovolemic shock. Upon admission, he was treated with intramuscular analgesics (dipyrone, 500 mg, and tramadol, 100 mg) and intravenous fluids.

The hemogram revealed neutrophilia and thrombocytopenia, while the thick smear was positive for *P. vivax* with 24 000 parasites per μl. During his brief 4-hour hospital stay, the patient experienced progressive deterioration of his general condition, culminating in a cardiopulmonary arrest that did not respond to resuscitation efforts, resulting in his death. Histopathological examination showed aggregates of polymorphonuclear neutrophils and plasmacytosis. The patient did not receive parenteral antimalarial treatment.

Splenic rupture associated with malaria is a severe and potentially fatal complication. Therefore, any painful splenomegaly in an acute malaria episode should be considered a warning sign of potential splenic rupture.

It is crucial to enhance the awareness of local medical teams regarding this complication, promoting timely diagnosis and treatment to improve clinical outcomes.

La malaria, o paludismo, es una enfermedad infecciosa causada por protozoarios del género *Plasmodium*, que se transmite a los humanos por la picadura de mosquitos hembra del género *Anopheles* infectadas con el parásito ^1^. Esta enfermedad presenta un espectro clínico que abarca desde las formas asintomáticas y los cuadros clínicos no complicados, hasta las complicaciones graves que pueden llevar a la muerte.

Generalmente, se presenta fiebre, escalofríos, sudoración, cefalea y malestar general. En los casos graves, puede haber compromiso de órganos vitales, lo cual incrementa significativamente la mortalidad. Entre las complicaciones sobresalen las hematológicas, las hepáticas y las renales. Una complicación inusual y potencialmente mortal es la ruptura esplénica ^2^.

El objetivo del presente estudio fue describir y analizar el curso clínico y fisiopatológico, y el pronóstico de un caso fatal de malaria complicada por *Plasmodium vivax*.

## Reporte de caso

Se trata de un paciente de sexo masculino, de 28 años, procedente y residente de El Retorno (Guaviare), que consultó el 15 de agosto del 2022 al servicio de urgencias del hospital por un cuadro clínico de tres días de evolución. En el momento de la consulta, el paciente manifestaba dolor toráxico, localizado en el lado izquierdo, de tipo punzada y sin irradiación, con una intensidad de 9/10. Además, refería fiebre no cuantificada, astenia y adinamia.

En la historia clínica no se consignaron comorbilidades, cirugías previas ni antecedentes de traumatismos. El paciente negó episodios previos de malaria o de otras infecciones prevalentes en la zona; reportó alergia a los antiinflamatorios no esteroideos (AINE). Cuatro días antes del ingreso, consultó al servicio de urgencias y se retiró voluntariamente sin valoración médica.

En la valoración inicial se encontró un paciente álgido, alerta, consciente, orientado en persona, tiempo y espacio con una escala de Glasgow de 15/15. Se registraron los siguientes signos vitales: tensión arterial de 122/88 mm Hg, frecuencia cardíaca de 160 latidos por minuto, frecuencia respiratoria de 24 respiraciones por minuto y temperatura de 36,5 °C; la saturación de oxígeno era del 97 %, y con talla de 173 cm, peso de 70 kg y un índice de masa corporal de 23,4 kg/m^2^.

En el examen físico, se evidenció dolor a la palpación localizado en el hemitórax izquierdo, sin irradiación. El abdomen se encontraba blando, depresible, sin dolor a la palpación ni visceromegalias. No se identificaron signos clínicos sugestivos de irritación peritoneal en ese momento. Se hizo la impresión diagnóstica inicial de síndrome de la articulación condrocostal izquierda.

Se le administró tratamiento analgésico intramuscular de 500 mg de dipirona cada 6 horas y 100 mg de tramadol cada 6 horas, además, líquidos intravenosos para mantenimiento. Asimismo, se ordenaron exámenes paraclínicos, que incluyeron gota gruesa, hemograma y electrocardiograma. Posteriormente, el paciente fue hospitalizado para seguimiento y manejo adecuado de su condición.

Durante la estancia hospitalaria, aproximadamente una hora después de su ingreso, el paciente presentó tres episodios de vómito con contenido gástrico y tres deposiciones líquidas. En ese momento, se encontraba pálido, en mal estado general, deshidratado y consciente; sus signos vitales fueron: presión arterial de 80/40 mm Hg, frecuencia cardíaca de 148 latidos por minuto, frecuencia respiratoria de 20 respiraciones por minuto, temperatura de 36,4 °C, saturación de oxígeno del 97 % y tensión arterial media de 53,3 mm Hg. Estos hallazgos llevaron al diagnóstico de choque hipovolémico, por lo que se inició el manejo con líquidos intravenosos.

Se recibieron los resultados de los estudios complementarios: en el electrocardiograma no hubo signos de isquemia, aunque se evidenció taquicardia sinusal con una frecuencia cardiaca de 140 latidos por minuto. El hemograma reveló un recuento de leucocitos de 9900 por μl, con el 72 % de neutrófilos, 94 500 plaquetas por μl y hemoglobina del 12,6 %. Además, en la gota gruesa se encontraron 24 000 parásitos de *P. vivax* por μl.

Cuatro horas después de su ingreso, el paciente presentó un deterioro significativo de su condición general. Se encontraba disneico y consciente, con un puntaje de Glasgow de 15/15. En la evaluación, se observó palidez acentuada con piel fría y húmeda. En ese momento, los signos vitales eran los siguientes: frecuencia cardiaca de 45 latidos por minuto (con tendencia progresiva a la baja), presión arterial de 80/40 mm Hg y tensión arterial media de 53,33 mm Hg, frecuencia respiratoria de 20 respiraciones por minuto, temperatura de 36,5 °C y saturación arterial del 98 %. La hipotensión persistente evolucionó a paro cardiorrespiratorio, lo que llevó a la realización de maniobras de reanimación. Aunque respondió inicialmente a las medidas de reanimación, el paciente falleció.

En el análisis histopatológico del bazo se observó pérdida de la arquitectura esplénica, con acentuada congestión vascular, hemorragia intraparenquimatosa y ruptura capsular; además, infiltrado neutrofílico y plasmocitosis reactiva, hallazgos concordantes con la ruptura esplénica hemorrágica (figura 1).

**Figura 1:**
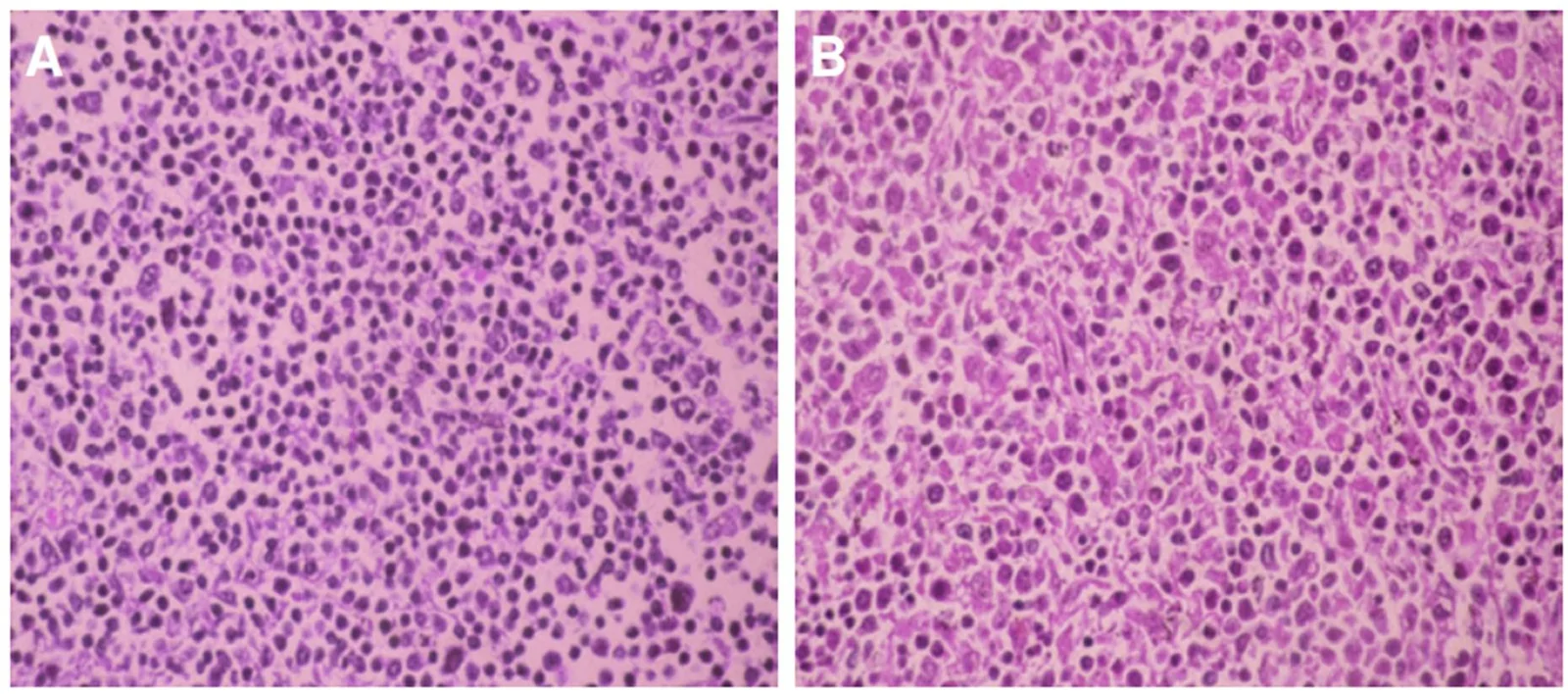
Bazo infectado por *Plasmodium vivax*. Se evidencia la presencia de agregados de neutrófilos polimorfonucleares y plasmocitosis reactiva asociada a sepsis de origen inespecífico. Hematoxilina y eosina, (A) 40X, (B) 100X.

Asimismo, se documentó necrosis tubular aguda en el riñón, congestión centrolobulillar en el hígado y edema alveolar difuso en el pulmón. El patrón multiorgánico es compatible con una lesión por hipoperfusión o choque en un cuadro de malaria complicada.

### Vigilancia y análisis de mortalidad

La unidad de análisis de mortalidad del departamento revisó la historia clínica, efectuó entrevistas al entorno y adelantó búsqueda activa comunitaria; fue realizada por el equipo de vigilancia epidemiológica conforme al protocolo nacional. Se documentó residencia en una zona rural de difícil acceso geográfico, ubicada en área endémica para malaria, y desplazamientos frecuentes del paciente a zonas de explotación agrícola. Tras la búsqueda activa en el núcleo familiar y comunidad, no se identificaron otros casos confirmados de malaria.

Con base en la información clínica y de laboratorio, junto con los hallazgos anatomopatológicos, el comité concluyó que la malaria complicada había sido la causa primaria de muerte.

### Aspectos éticos

El presente reporte de caso no implicó intervención ni experimentación en seres humanos y se desarrolló conforme a los principios éticos establecidos en la Resolución 8430 de 1993 y la Ley 23 de 1981 del Ministerio de Salud de Colombia. Por lo tanto, no requirió aprobación de un comité de ética institucional. Se obtuvo el consentimiento informado por escrito de la esposa del paciente fallecido para el uso académico y la publicación del caso y de las imágenes que lo acompañan. Se adoptaron todas las medidas necesarias para garantizar la confidencialidad de la información clínica, sin incluir datos ni elementos que permitieran la identificación del paciente.

## Discusión

Este reporte describe un caso de ruptura esplénica patológica con desenlace fatal asociada a malaria complicada por *P. vivax* en el departamento del Guaviare. La malaria causada por *P. vivax* es la forma clínica predominante en Colombia y representa, aproximadamente, el 60 % de los casos registrados a nivel nacional ^3^.

Las formas complicadas de malaria son eventos de baja frecuencia en Colombia, cuya incidencia estimada es menor del 2 % del total de casos; de estos, cerca del 65 % son producidos por la especie *P. vivax*^3^. A su vez, la ruptura esplénica por esta causa se destaca como una de las complicaciones más raras. Sin embargo, en las series reportadas de ruptura patológica del bazo a nivel global, la malaria es una de las causas principales ^4^.

Durante las primeras décadas del siglo XX, en el contexto de la terapia malárica, se reportaron desde varias decenas hasta algunos cientos de casos de ruptura esplénica relacionada con esta enfermedad, estimándose una incidencia aproximada de 1,2 por cada 1000 casos. Hasta la década de 1960, se habían documentado cerca de 200 casos a nivel mundial; un tercio de estos estaban asociados a la «malarioterapia», que consistía en la inducción terapéutica de infecciones maláricas para el tratamiento de la neurosífilis, antes de la introducción de la penicilina. Sin embargo, la incidencia actual de la ruptura esplénica por malaria sigue siendo incierta debido a la falta de estudios prospectivos, la pobre detección y el probable subregistro ^5^^,^^6^.

La ruptura esplénica espontánea se sustenta en cuatro criterios clínico- patológicos: (i) ausencia de traumatismo o maniobras iatrogénicas, (ii) esplenomegalia reactiva o inflamación aguda, (iii) hemorragia intraperitoneal o hematoma subcapsular, y (iv) confirmación anatomopatológica (congestión, hemorragia intraparenquimatosa y ruptura capsular) ^7^^,^^8^.

En este paciente se documentó la ausencia de trauma y se informaron hallazgos anatomopatológicos sugestivos de ruptura esplénica. La presentación fue atípica, con dolor en el hemitórax izquierdo sin dolor abdominal ni irritación peritoneal al ingreso; posteriormente, evolucionó abruptamente a choque hipovolémico fatal. El dolor torácico puede explicarse por irritación diafragmática con dolor referido.

Teniendo en cuenta el curso clínico de la enfermedad, es probable que inicialmente el paciente se encontrara en un estado de choque compensado, en el que los mecanismos compensatorios del cuerpo, como la taquicardia y la taquipnea, mantuvieron una perfusión adecuada a los órganos vitales, sin mostrar signos evidentes de hipotensión ^9^^,^^10^. No obstante, con el avance de la enfermedad, estos mecanismos comenzaron a fallar, lo que resultó en hipotensión persistente, dificultad respiratoria y signos de disfunción orgánica que, finalmente, generaron un choque grave. Esta complicación se ha descrito como criterio de malaria complicada según la Organización Mundial de la Salud (OMS) ^11^.

La ruptura esplénica probablemente ocurrió durante la hospitalización; diversos factores pueden haber contribuido a su presentación. Se ha sugerido que uno de los posibles factores desencadenantes está relacionado con la presión y la contracción que ejercen los músculos abdominales sobre el bazo durante el vómito, la tos o la defecación, entre otras. En este caso, el paciente experimentó tres episodios de emesis y de evacuaciones, lo que habría favorecido la ruptura esplénica ^4^^,^^5^.

Otras posibles causas de esta complicación incluyen el aumento de la tensión interna en el bazo, como resultado de la proliferación excesiva de células y la sobrecarga sanguínea en el órgano. Además, la obstrucción de los vasos sanguíneos debido a la proliferación anómala de células reticuloendoteliales puede llevar a trombosis e infarto esplénico. Este proceso favorece la acumulación de sangre en los espacios intersticiales y bajo la cápsula esplénica, lo que provoca hemorragias adicionales y la separación progresiva de la cápsula. A medida que la cápsula se distiende, eventualmente cede, lo que lleva a la ruptura del bazo ^4^^,^^5^. Sin embargo, el estudio histopatológico de este caso no evidenció signos de infarto esplénico.

Se ha demostrado que *P. vivax* tiene una mayor afinidad por los reticulocitos jóvenes. Además, la pulpa esplénica desempeña un papel fundamental en la maduración de estos reticulocitos, así como en el crecimiento y la replicación del parásito ^12^. En este contexto, se ha propuesto la existencia de un ciclo de vida intraesplénico críptico en las infecciones por *P. vivax*^12^^,^^13^. Por lo tanto, la malaria causada por esta especie debe considerarse principalmente como una infección del bazo, que actúa como un nicho fisiológico natural para la maduración de los reticulocitos inmaduros ^13^.

La trombocitopenia es una alteración hematológica frecuente en malaria, pero las manifestaciones hemorrágicas son poco comunes, y la relación entre el recuento plaquetario y el sangrado clínico no es constante ^14^^,^^15^. En las series publicadas, incluso los recuentos muy bajos pueden cursar sin sangrado manifiesto (por ejemplo, 9000 plaquetas por μl), y no se ha demostrado una asociación contundente entre la trombocitopenia y la esplenomegalia, lo que apunta hacia mecanismos inmunológicos o periféricos más que a secuestro mecánico como explicación principal ^13^.

En este caso (94 500 plaquetas por μl) no es posible atribuir causalidad entre la trombocitopenia y la ruptura esplénica. El recuento bajo pudo haber limitado la hemostasia una vez instaurada la hemorragia, sin constituir por sí mismo un factor desencadenante del evento.

En un estudio se encontró que la mayoría de los casos de ruptura esplénica se había presentado en adultos, con una notable subrepresentación de niños, posiblemente debido a la mayor elasticidad de la cápsula esplénica en los individuos más jóvenes, que puede ofrecer una mayor resistencia a la rotura. La edad media de los pacientes con ruptura esplénica fue alrededor de los 32,1 años, y un número significativo de ellos eran varones ^4^.

En otro estudio se destaca que la ruptura esplénica durante la malaria es una complicación grave, con una tasa de mortalidad del 22 % entre los casos revisados. El riesgo de muerte fue significativamente mayor en los casos de *P. falciparum* que en los de *P. vivax*^5^.

Aunque tradicionalmente se ha considerado que la malaria por *P. vivax* presenta menos manifestaciones clínicas graves en comparación con los de P. falciparum, en Colombia se observa lo contrario ^3^^,^^16^. La OMS define la malaria complicada por *P. vivax* de manera similar a la malaria por *P. falciparum*, sin establecer umbrales específicos de parasitemia ^9^. En el país, se han reportado niveles de parasitemia de *P. vivax* con una media geométrica de 5815 parásitos por μl (IC_95_%: 5 428 a 6 230) en los casos de malaria complicada ^17^. En este caso, se observaron niveles de parasitemia cuatro veces superiores a los niveles esperados, pero es incierto si esto pudo haber contribuido al desenlace.

En la ruptura esplénica, la prioridad es la estabilización hemodinámica inmediata con acceso de alto flujo, cristaloides titulados y hemoderivados para corregir la coagulopatía ^18^. La ecografía FAST (*Focused Assessment with Sonography for Trauma*) confirma el hemoperitoneo sin retrasar el control del sangrado en los pacientes inestables.

La decisión debe ser temprana: manejo no operatorio -solo con estabilidad sostenida y monitorización estrecha- o esplenectomía urgente, ante la inestabilidad o sangrado no controlado. Cuando se dispone de los recursos necesarios, la oclusión selectiva de la arteria esplénica mediante técnicas de radiología intervencionista constituye una alternativa terapéutica. En paralelo, se debe iniciar la administración intravenosa de artesunato y completar el esquema oral según las guías ^19^. En el presente caso, el paciente no alcanzó a recibir la terapia parenteral indicada.

La unidad de análisis de mortalidad confirmó la malaria complicada como la causa primaria de muerte y no encontró agregación de casos en el entorno inmediato del paciente, en concordancia con la investigación epidemiológica de campo.

Entre las limitaciones de este reporte se encuentra la necesidad de consolidar la información procedente de múltiples fuentes clínicas para reconstruir la evolución del caso. A pesar de ello, la información recopilada permitió establecer de forma constante la secuencia de acontecimientos clínicos, diagnósticos y terapéuticos relevantes.

Entre las lecciones aprendidas que nos deja el caso podemos resaltar que:

- La ruptura del bazo por malaria es una complicación grave y potencialmente mortal.
- Cualquier esplenomegalia dolorosa durante un caso agudo de malaria debe interpretarse como un signo de alarma temprano de ruptura esplénica.
- Se debe mejorar la percepción por parte de los equipos médicos locales sobre esta complicación, de modo que conduzca a un diagnóstico y tratamiento oportunos, lo cual mejoraría el pronóstico del paciente y contribuiría a la reducción de la letalidad asociada a la malaria complicada.
- Es necesario caracterizar y vigilar estrechamente las complicaciones asociadas a la malaria complicada por *P. vivax* para poder generar evidencia clínico-epidemiológica fidedigna y confiable que permita mejorar la detección y comprensión de otras manifestaciones infrecuentes de compromiso de órganos. Esto podría contribuir a esclarecer la patogénesis de la malaria complicada por *P. vivax* y su potencial impacto en la salud pública.
- Se recomienda revisar la definición de caso de malaria complicada por P. vivax y tener en cuenta los umbrales de parasitemia.
- El diagnóstico temprano y el tratamiento oportuno de esta emergencia requieren un enfoque multidisciplinario y una actuación rápida, debido a las altas tasas de morbimortalidad que causa.

En conclusión, este reporte describe la defunción por malaria complicada causada por *P. vivax*, asociada a choque hipovolémico secundario a ruptura esplénica e hiperparasitemia en un adulto joven residente de un área endémica del país. Este caso destaca el potencial de *P. vivax* para ocasionar complicaciones graves y potencialmente fatales, así como la importancia de su reconocimiento y manejo oportunos.
